# Power and Time Dependent Microwave Assisted Fabrication of Silver Nanoparticles Decorated Cotton (SNDC) Fibers for Bacterial Decontamination

**DOI:** 10.3389/fmicb.2017.00330

**Published:** 2017-03-03

**Authors:** Abhishek K. Bhardwaj, Abhishek Shukla, Rohit K. Mishra, S. C. Singh, Vani Mishra, K. N. Uttam, Mohan P. Singh, Shivesh Sharma, R. Gopal

**Affiliations:** ^1^Centre for Environmental Science, University of AllahabadAllahabad, India; ^2^Laser Spectroscopy and Nanomaterials Lab., Department of Physics, University of AllahabadAllahabad, India; ^3^Centre for Medical Diagnostic and Research, Motilal Nehru National Institute of TechnologyAllahabad, India; ^4^High-Intensity Femtosecond Laser Laboratory, The Institute of Optics, University of RochesterRochester, NY, USA; ^5^Centre of Biotechnology, University of AllahabadAllahabad, India

**Keywords:** silver nanoparticles decorated cotton (SNDC) fibers, microwave irradiation, SNPs functionalized textile, antibacterial efficacy, cell death, ROS

## Abstract

Plasmonic nanoparticles (NPs) such as silver and gold have fascinating optical properties due to their enhanced optical sensitivity at a wavelength corresponding to their surface plasmon resonance (SPR) absorption. Present work deals with the fabrication of silver nanoparticles decorated cotton (SNDC) fibers as a cheap and efficient point of contact disinfectant. SNDC fibers were fabricated by a simple microwave assisted route. The microwave power and irradiation time were controlled to optimize size and density of silver nanoparticles (SNPs) on textile fibers. As prepared cotton fabric was characterized for ATR-FTIR, UV-VIS diffuse reflectance, SEM and TEM investigations. Size of SNPs as well as total density of silver atoms on fabric gets increased with the increase of microwave power from 100 W to 600 W. The antibacterial efficacy of SNPs extracted from SNDC fibers was found to be more effective against Gram-negative bacteria than Gram-positive bacteria with MIC 38.5 ± 0.93 μg/mL against *Salmonella typhimurium* MTCC-98 and 125 ± 2.12 μg/mL against *Staphylococcus aureus* MTCC-737, a linear correlation coefficient with *R*^2^ ranging from ∼0.928–0.935 was also observed. About >50% death cells were observed through Propidium Iodide (PI) internalization after treatment of SNPs extracted from SNDC fibers with concentration 31.25 μg/mL. Generation of ROS and free radical has also been observed which leads to cell death. Excellent *Escherichia coli* deactivation efficacy suggested that SNDC fibers could be used as potentially safe disinfectants for cleaning of medical equipment, hand, wound, water and preservation of food and beverages.

## Introduction

Silver nanoparticles (SNPs) are extensively employed in healthcare and pharmaceutical products that include coating material for medical devices, orthopedic or dental graft materials, air/water filters, food containers, topical aids for wound repair, clothing and textile fabrics (Piccinno et al., 2012; Bendi et al., 2015). Since ancient time silver is being used as an effective antimicrobial agent, the characteristic feature that has been accredited to SNPs with the advent of nanotechnology enhanced its antimicrobial activities by several orders (Davies and Etris, 1997; Swarnkar et al., 2016; Mahmoud et al., 2016). However, increasing interaction of SNPs with human and the environment has been an important issue of concern as it has been found to cause several undesired problems for the later. The NPs could migrate from textile to human sweat and increase its exposure to skin affecting human physiology (Windler et al., 2012). Their release into waterways could adversely affect the aquatic life (Mahmoudi and Serpooshan, 2012). Also, high surface energies of SNPs tend to agglomerate resulting in application difficulties (Grumezescu et al., 2013). This is an important element to immobilize SNPs on a matrix system that would allow an efficient and effective disinfectant by preventing its aggregation and thus reducing its migration plausible threat to the environment (Agnihotri et al., 2013; Zafar et al., 2014).

Researchers have rigorously employed flexible substrates such as fabrics, plastics, textiles, and papers for NPs immobilization. SNPs specifically have been incorporated into a range of cellulosic materials, such as bacterial cellulose, cotton fabric, filter paper, and cellulose gels (He et al., 2003; Maneerung et al., 2008; Ferraria et al., 2009; Dankovich and Gray, 2011; Bendi and Imae, 2013). In this context, cotton owing to its natural softness, permeability, high moisture absorbency, mechanical strength, renewable and heat retaining properties (Ravindra et al., 2010; Tang et al., 2012) has been recently employed as the most successful material. The super-hydrophobic surface on cotton fabric guarantees its dryness and cleanness which are considered as desired features, in particular on its outside facet (Hoefnagels et al., 2007; Xu and Cai, 2008; Bae et al., 2009; Gao et al., 2009; Hao et al., 2010; Xu et al., 2010; Berendjchi et al., 2011). Cotton fabric is an ideal place for settling and growing pathogenic bacteria because of its porous and hydrophilic structure.

To prepare antimicrobial silver-treated cotton fabrics, most of the recent research activities are concerned on preparations of SNPs with controlled size and developing routes to impart SNPs to cotton fabrics. Traditionally, in preparing SNP, numerous reducing agents, such as sodium borohydride (NaBH_4_), formaldehyde, sodium citrate, hydrazine, ascorbic acid, glucose and γ-ray or UV irradiation, were utilized to reduce the silver cations, while some polymeric materials, such as poly(vinylpyrrolidone) (PVP), poly(ethylene glycol) (PEG), and some surfactants were used as stabilizers to prevent NPs agglomeration and precipitation (Luo et al., 2005; Stevenson et al., 2012). In addition, during the process of antimicrobial finishing on cotton fabrics with SNPs, a binders, such as dimethyloldihydroxyethylene urea (DMDHEU), polyurethane resin, poly(2-aminoethyl methacrylate) (PAEMA) Polyacrylic esters (PALS), and PDDA is required to fix the SNPs on the fibers to provide durability of antimicrobial properties (Zhang et al., 2009; Liu et al., 2014). Nevertheless, the synthesis of a monodisperse and stable SNPs suspension is challenging and may go through tedious and complex procedures, which may hinder the practical applications of SNPs on textiles. The size of the SNPs can be controlled by the concentration of silver nitrate and reducing agent, temperature, and duration of reaction (Dankovich, 2014; Liu et al., 2014).

In this paper, we report *in situ* fabrication of SNPs impregnated cotton fiber by domestic microwave irradiation. The bactericidal efficacy of the SNDC fibers was tested against both Gram positive and Gram negative bacteria. This study reveals that the SNDC fibers might be successfully employed to small scale system for point of use water decontamination, surface sterilization of medical or other equipment, and wound healing. This is a cheap, portable, eco-friendly, and point of use system for contact killing of microbes and disinfection of drinking water.

## Materials and Methods

### Procurement of Chemicals and Culture Media

Analytical grade chemicals such as Silver nitrate (AgNO_3_ 99.98%), trisodium citrate (∼99%) and poly (diallyldimethyl ammonium chloride) (PDDA) (M_w_ = 200000–350000 g.mol^-1^), and Milli-Q grade water were purchased from Merk, medical cotton and cotton bandage (made Krishna Handloom Pvt. Ltd, India) as well as nutrient agar (NA), Mueller Hinton Broth (MHB) and Mueller Hinton Agar (MHA) were procured from Himedia Pvt. Ltd for antibacterial assay. For cell permeability propidium iodide (PI) was purchased from Sigma Aldrich, Pvt. Ltd.

### Selected Bacterial Pathogens

Three bacterial pathogens *Escherichia coli* (MTCC-723); *Staphylococcus aureus* (MTCC-737) and *Salmonella typhimurium* (MTCC-98) were procured from microbial type culture collection (MTCC), Chandigarh, India. The cultures were maintained on NA slants at 4°C throughout the study used as stock.

### Fabrication of Silver Nanoparticles Decorated Cotton (SNDC) Fibers

**FIGURE 1:**
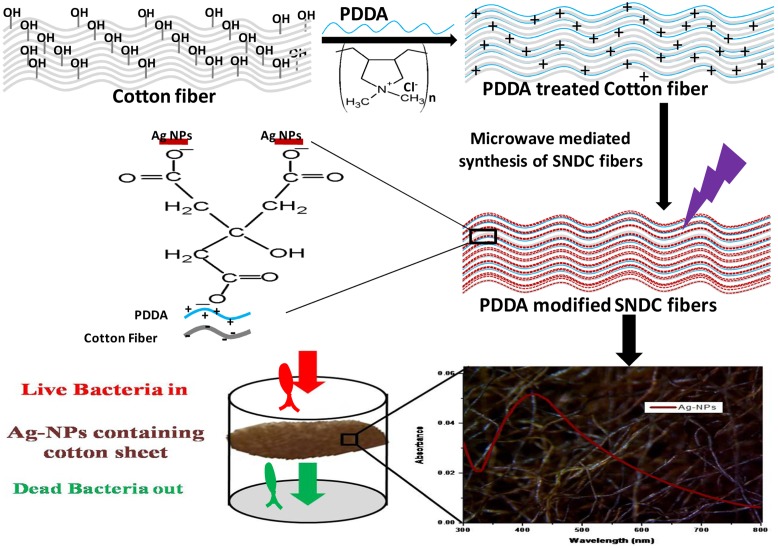
**Illustration showing microwave assisted fabrication mechanism of PDDA modified silver nanoparticles decorated cotton (SNDC) fibers**.

In a typical synthesis procedure, cotton pieces (7 cm × 7 cm) were dipped in boiling double distilled water for 4 h to remove impurity and were transferred into the oven at 50°C for 6 h for drying. This cleaned cotton was immersed into the aqueous solution of 2.5 M PDDA for 12 h and dried at room temperature. After that PDDA modified cotton soaked in 2 M trisodium citrate. Wet cotton pieces were squeezed gently for the removal of extra trisodium citrate followed by addition of 6 ml, 0.01 M aqueous silver nitrate solution. For microwave assisted decoration of cotton fiber by SNPs, they were placed in domestic microwave oven (Sharp model no MW73V/XT, 2.45 GHz, 800 W) with rotating disk. The density of SNPs loaded on fiber and their sizes were varied with time (60–180 s) and power (100–600 W) of microwave irradiation. In order to avoid over heating/burning of cotton, the microwave was switched off after every 60 s of irradiation. In order to remove excess un-reacted silver precursor and loosely bound SNPs, treated cottons were kept in warm water for 2 h followed by drying in hot air oven at 45°C for 8 h. A mechanism for the fabrication of SNDC fibers is depicted in (**Figure 1**).

### Characterization of Silver Nanoparticles Decorated Cotton Fibers

Qualitative imaging of SNDC fiber samples was done by standard photography and microscopy (magnification 200 X by digital microscope) which show the change in color from white to yellow/orange, an indication of SNPs adsorbed on the surface of the cotton fiber. The density of SNPs on cotton fibers was measured from diffused UV-VIS reflectance spectra recorded using PerkinElmer Lambda 35 double beam spectrophotometer equipped with Labsphere RSA-PE-20 diffused reflectance accessory with barium sulfate as white standard. ATR-FTIR spectra of SNDC fiber samples were recorded using ABB MB3000 series FTIR spectrometer (ABB, Bomem Inc. Canada), Tecnai G2-20 (FEI Company, Netherland), high resolution transmission electron microscope (HRTEM) operating at 200 kV and field emission scanning electron microscope (FESEM, Nova NanoSEM 450 series)was used for size and shape measurements of as produced SNPs.

### Extraction of Silver Nanoparticles from SNDC Fibers

PDDA polymer is responsible to provide good binding affinity between cotton fibers and SNPs. We used normal SNDC fibers (PDDA untreated SNDC) for SNPs extraction, due to its lower binding affinity with SNPs and higher particle release capacity in compare to PDDA treated SNDC fibers. PDDA untreated cotton were used to prepare SNDC (microwave irradiation 600 W, 1 min) fallowed by their transfer into 100 mL warm deionized water (70°C) and continuous shaking for four hrs consequently SNPs was release in the water medium and form colloidal SNPs. Extracted colloidal solution of SNPs were used for UV-VIS absorption, TEM imaging and flowcytometry investigations.

### Antibacterial Susceptibility of Extracted SNPs

Silver nanoparticless extracted from SNDC fibers were used for determining antibacterial activity as per broth microdilution method described by Mishra et al. (2016a). Briefly, SNPs solution extracted from the SNDC fibers is used as stock solution with 20 mg/ml, which was then serially diluted 1:10 in the medium in order to attain final concentration ranging from 500 to 3.9 μg/mL. Each well was subsequently filled with 100 μl of inoculum. The initial concentration of inoculum was 1 × 10^6^ cells/ml (adjusted according to 0.5 McFarland) in MHB. The plates were stored at 35 ± 2°C in a wet chamber for 24 h and experiments were conducted in triplicate.

### Quantification of Bacterial Growth

After 24 h of incubation, the optical density of the microtiter plates was recorded spectrophotometrically at 492 nm using SpectraMaxplus^384^ (Molecular Devices, USA). The changes in OD over time were used to generate growth curves at each drug concentration against the control. The normalized OD of the SNPs treated wells (OD obtained after subtraction of the background OD) was used for the generation of turbidimetric growth curves. Percentage of growth inhibition at each drug concentration was calculated using the formula:

(1) $$\mathrm{Growth inhibition}(\%)\;=\frac{{\mathrm{OD}}_{492}\,\;\mathrm{of wells containing the Ag NPs soln.}}{{\mathrm{OD}}_{492}\,\;\mathrm{of well without treatment}}\times 100$$

### Determination of Minimum Inhibitory Concentrations (MICs)

Minimum inhibitory concentrations were determined spectrophotometrically using the software SoftMax^®^ Pro-5 (Molecular Devices, USA). For the SNPs solution, the MIC was determined as the lowest nanoparticle concentration showing ≥70% growth inhibition compared with the growth in the treatment-free well. Each test was performed in triplicates.

### Determination of Minimum Bactericidal Concentration (MBC)

Minimum bactericidal concentration was defined as the lowest concentration of the SNPs at which 99.99% or more of the initial inoculum was killed. Hundred microliter aliquot of inoculum was taken aseptically from those wells that did not show turbidity and it was poured on MHA plates followed by incubation for 24 h at 35 ± 2°C. The absence of growth reflected that the concentration was lethal. The number of surviving organisms was determined by viability counts. All tests were performed in triplicates.

### Flowcytometric Analysis for Plasma Membrane Permeabilization and ROS Detection

The measurement of membrane permeability of selected bacterial pathogens treated with SNPs were observed through flowcytometer using fluorescence dye i.e., PI and Protein leakage. The selected bacterial inocula 1 × 10^6^cells/ml were prepared and adjusted according to 0.5 McFarland. Each bacterial cell suspension was incubated with various concentrations 15.62 and 31.25 μg/mL of SNPs for 24 h at 35 ± 2°C. Following incubation, cells were washed and resuspended in PBS and subsequently stained with PI (20 μg/ml) for 20 min in a dark chamber. The cells were analyzed by BD Accuri C6 (Becton Dikinson, San Jose, CA, USA). Intrinsic parameter (SSC-A) and fluorescence in FL-2 channel for PI were acquired and recorded over the logarithmic scale. The changes in treated cells were compared with untreated cells as well as with cells treated with SNPs.

For ROS detection, the bacterial cell pellet was suspended in a LB broth and incubated with DCFH-DA reagent at 37°C for 30 min in the dark. The fluorescence was measured as above at an excitation wavelength 485 nm and emission wavelength 528 nm. Protein leakage was analyzed in culture supernatant using Bradford assay as per the manufacturer’s protocol. Ten microliter supernatant from each bacterial culture was transferred to 96 well plates followed by adding 250 μl of Coomassie Blue reagent. After mixing the plate on a plate shaker for 30 min and further incubation at room temperature for 10 min, the absorbance was measured at 595 nm using a spectrophotometer (SpectraMaxplus^384^, Molecular Devices, USA). BSA was used as the standard for which a standard curve was drawn at each experiment to determine the protein concentration for each sample.

### Antibacterial Efficiency of Silver Nanoparticles Decorated Cotton Fibers

The antibacterial susceptibility of SNPs-cotton and pure cotton fiber were carried out by disk diffusion assay. In this assay, *E. coli*, *S. aureus* and *S. typhimurium* were selected as the model bacteria. The microorganisms were cultured overnight at 37°C in NA. The final cell concentrations of bacterial inoculants were 10^6^–10^7^ colony forming unit (CFU)/ml. The fabricated samples were cut down into small pieces (0.5 cm × 0.5 cm) and delivered on the agar plates, incubated at 37°C for 24 h, for susceptibility study using modified disk diffusion assay technique (Hu et al., 2009; Ravindra et al., 2010). The culture plates were observed for the presence of the circular zone of bacterial growth inhibition/clear zone around the SNDC fibers, expressed in terms of the average diameter of the zone of inhibition in millimeters.

### Filtration Efficacy SNDC Fibers and Leaching of Ag^+^ Ions

The antibacterial efficacy of SNDC fibers was tested against *E. coli* (MTCC 723) because it is widely accepted as an indicator of fecal contamination in potable water. Cotton fiber with a thickness of 0.4 cm was used as a control and the same thickness of cotton fibers were fabricated with exposure to microwave irradiation (600 W) for 1 min.

The inocula suspension of *E. coli* was prepared and adjusted according to 1 × 10^8^ CFU/mL in liquid media. This suspension was used as a model of contaminated water which permeates through SNDC fibers at the rate of 100 mL within 8 min. A small amount of *E. coli* bacteria were retained in the cotton filter and almost passed through SNDC fibers. These isolated bacteria from the effluent were centrifuged and analyzed for viability. The qualitative re-growth experiments were performed in MHB. Selected bacterial growth kinetics was analyzed with an optical density at 492 nm for every 2 h as compared with the positive control. Further, to identify bactericidal efficacy of SNDC fibers the effluent was placed on MHA and incubated overnight at 37°C for 24 h for observation of bacterial colonies. The Ag^+^ ions concentration leached from SNDC based filter in the disinfection process was analyzed using Inductively Coupled Plasma Mass Spectrometer (ICP-MS) (Thermo Fisher Scientific, Germany).

### Statistical Analysis

All experiments were independently repeated in triplicates. Results were expressed as mean ± standard error (SE). The statistical analysis was performed in MS Excel and Origin 6.1 software.

## Results and Discussion

Microwave irradiation is a one of the most promising method for rapid and green synthesis of SNPs (Raveendran et al., 2006; Dankovich, 2014). The SNPs were readily formed on cellulosic cotton sheet by microwave irradiation of the cotton saturated with precursor and reducing agent. This reaction does not occur at room temperature. Here reduction reaction catalyzed by the microwave irradiation results reduction of silver ions (Ag^+^) into silver neutrals (Ag^0^), which get start nucleation to form SNPs on the PDDA modified surface of cotton fibers to reduce their surface energy. Power and time of microwave irradiation are varied in order to increase density of SNPs on the surface of cotton fibers.

### UV-Visible Diffuse Reflectance Spectroscopy for the Investigation of Loading Density of SNPs on Cotton Fibers

**FIGURE 2:**
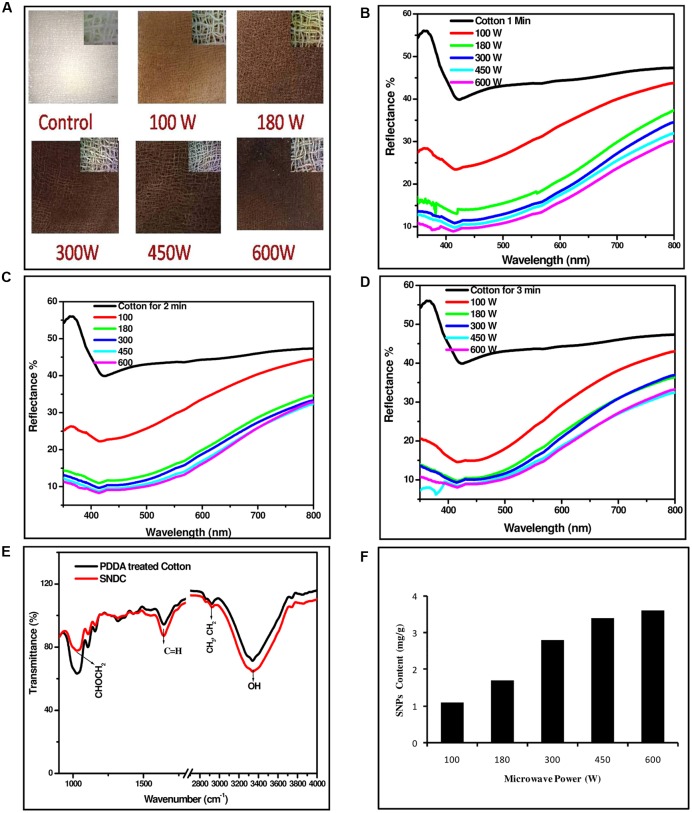
**Silver nanoparticles decorated cotton sheets prepared at different time and power of microwave irradiation, (A)** SNDC fibers prepared at 100–600 W for 3 min microwave irradiation, **(B)** UV-VIS diffuse reflectance spectra of SNDC at 1 min, **(C)** 2 min, **(D)** 3 min, **(E)** ATR-FTIR spectra cotton and SNDC fibers, **(F)** Content of SNPs on different SNDC sheets.

Color of cotton sheet soaked with aqueous solution of silver precursor changed from white to yellow/orange and finally brown with the increase in time of microwave irradiation at constant power or with the raise of power for given time. Change in the color of SNDC fibers can be directly correlated with the amount of SNPs loaded on them. UV-VIS diffuse reflectance spectra of SNDC fibers samples with variations in time of irradiation and microwave power are illustrated in (**Figure 2**). Valley of the reflectance spectra in the range of 430–450 nm is due to the out plane quadrupole plasmon resonance and some partial color change are also due to localized surface plasmon resonance (LSPR) (Kelly et al., 2003; Sherry et al., 2006; Shopa et al., 2010; Dankovich and Gray, 2011; Tang et al., 2012; Dankovich, 2014).

**FIGURE 3:**
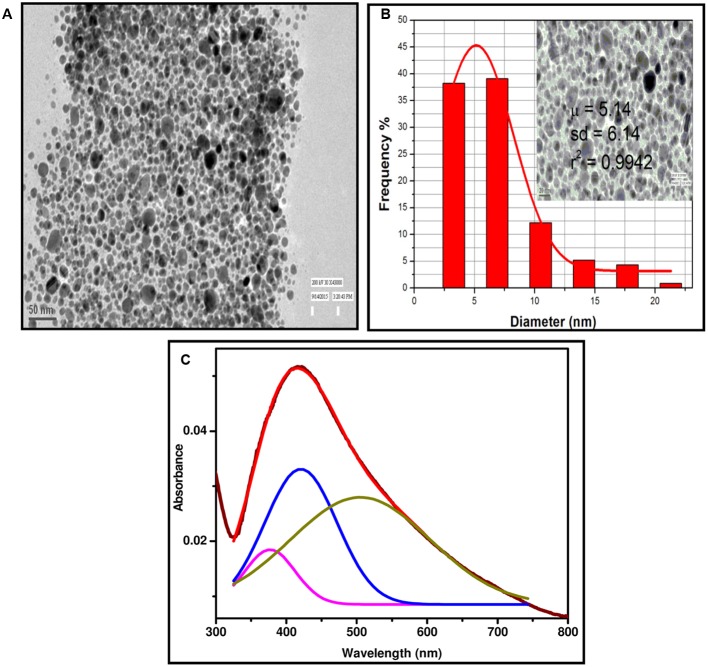
**(A)** TEM image of extracted SNPs from SNDC fibers, **(B)** Histogram represented distribution of SNPs diameters (inset, scale bar, 20 nm), **(C)** UV-VIS spectrum of solution of SNPs extracted from SNDC fibers.

UV-VIS diffuse reflectance spectra of SNDC fiber sheets fabricated with varying microwave power from 100 to 600 W for 1, 2, and 3 min of irradiations are shown in (**Figures 2B–D**). Optical photographs of undecorated and SNDC fiber at 100–600 W microwave irradiance for 3 min of irradiation are shown in (**Figure 2A**) with corresponding microscopic (20X) images in the inset. With the increase of microwave power or time of irradiation, reflectance in the complete spectral range decreases, which is an evidenced of increase in the density of SNPs on cotton fiber. Decrease in the reflectance is more pronounced at ∼450 nm corresponding to the SPR absorption of silver. Reflectance corresponding to SPR decreases, while its position slightly shifted toward longer wavelength side with the increase of power or time of irradiation, which shows that density as well as size of the NPs increases with the increase in power or time of microwave irradiation (Tang et al., 2012). Consistent color change observed by necked eye further increase in the depth of valley at 450 nm in the reflectance spectra state that loading of SNPs increases with time as well as with power of microwave irradiation. This is further elaborated by calculated SNP content of SNDC fibers shown in **Figure 2F**. Presence of SNPs on the cotton fiber is verified by spectroscopic as well as microscopic investigation. UV-VIS absorption spectrum of colloidal solution of NPs obtained by washing of an SNDC fibers sample prepared by microwave irradiation (600 W, 1 min) of PDDA untreated cotton fiber has intense SPR peak at 418 nm (**Figure 3C**), which proves presence of SNPs on cotton fibers. Same extracted colloidal solution has been used for TEM investigation and flowcytometry. SPR absorption spectrum, deconvoluted into three Gaussian peaks centered at 377, 421, and 505 nm, shows trimodal distribution of SNPs, which is also verified by TEM (**Figure 3C**). The variations of thermodynamic environment like temperature are responsible to regulate morphology and stability of SNPs (Agnihotri et al., 2014; González et al., 2014). In this study interaction of water and microwave radiation creates high temperature with increasing the power may cause quick water evaporation from aqueous solution. In case of increasing power of microwave, SNPs loading was greater on cotton fiber but SPR peak was broaden which suggest wide particle size distribution. This broadening is due to quick synthesis of SNPs at higher temperature catalyzes the rate of reaction and faster growth of nucleation takes place at different layer of cotton fiber. The same tendency was found in (**Figures 2B–D**). However, the cellulose polymer begins to degrade at high temperatures (Madorsky et al., 1958) and SNPs formation at elevated temperatures lead to a weaker structure for the cotton filter, which shorten its lifespan as a water filter.

Here limited concentration of reducing agent and precursor are taken which is responsible to stop reaction automatically when reaction was complete. However, nucleation and growth of NPs continued. During observation of defuse reflectance we found that there is no SPR peak shift at particular power and different time, it is because of only continuous loading of SNPs on the surface of cotton fiber. This is confirmed by the spectra of different time of irradiation with observed dip SPR.

### Fourier-Transform Infrared Spectroscopy for the Diagnosis of Interaction between Cotton Fiber and SNPs

Fourier-transform infrared (FTIR) spectra of SNDC fibers in attenuated total reflectance (ATR) mode, illustrated (**Figure 2E**), have a broad band corresponding to the stretching frequency of the hydroxyl group around 3400 cm^-1^ and a peak around 1642 cm^-1^ corresponds group of the cotton fiber (Mahadeva et al., 2014), while peaks at 1028, 1106, 1156, and 1427 cm^-1^ can be identified as a characteristic peak of cotton/cellulose (Fakin et al., 2012; Peng et al., 2016). The three vibration peak of C-O locate within the spectral range of 1200–1000 cm^-1^ are 1156 cm^-1^, 1106 cm^-1^, and 1028 cm^-1^ suggest cellulosic content of cotton. The spectral peak observed at 2925 (CHn), 1642 (C = C) and 1427 cm^-1^ (CH_2_) are characteristics of PDDA (Ding et al., 2014). The characteristic peaks position of PDDA treated cotton fiber after silver fabrication lying at 1642 cm^-1^ (C = C) and 1427 cm^-1^ (CH_2_) are shifted (Xu et al., 2014). This shifting is with respect to their original value in cotton, clearly indicates the existence of SNPs over surface of PDDA modified cotton fiber. Pure cotton has less transmittance than decorated cotton at certain above given peaks. The intensities of the peaks is lower for pure cotton than decorated cotton fabrics indicating the hydroxyl groups of cellulose before silver coating, whereas after SNPs loading the intensities of the related peaks are increased. This might be due to decrease of this group on the cotton fiber surface. The decreasing of hydroxyl groups may demonstrate physical adsorption of silver ions to these groups (Nourbakhsh and Ashjaran, 2012).

### TEM and SEM for Size, Shape and Distribution Measurements

**FIGURE 4:**
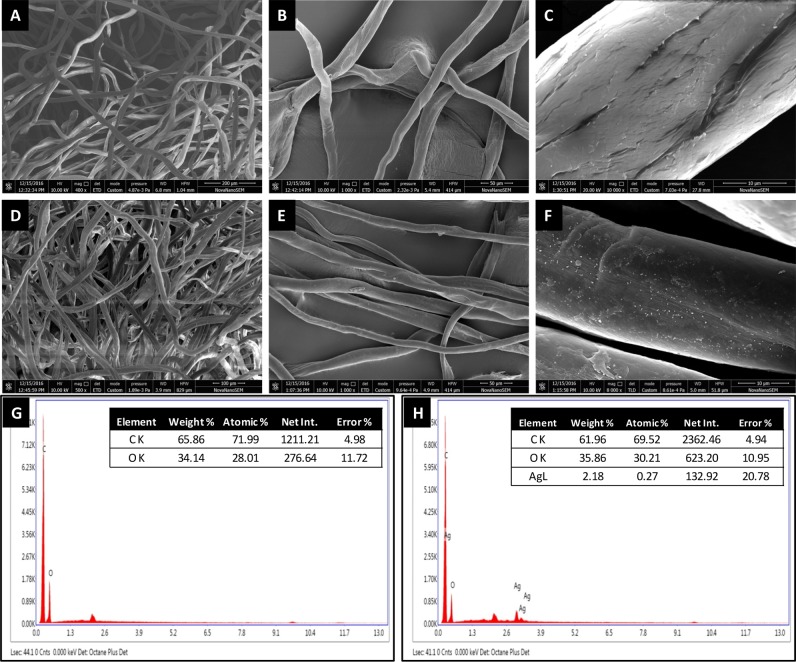
**Scanning electron micrograph (SEM) of SNDC fibers with different magnification (A–C)** normal cotton fibers **(D–F)** SNDC fibers, **(G,H)** EDX of normal cotton fibers and SNDC fibers.

Colloidal aqueous extracted SNPs synthesized by microwave-assisted reduction of SNPs over PDDA unmodified cotton has been taken for HRTEM characterization. The almost small, spherical, and monodispersed SNPs particles are shown in (**Figure 3A**), the average particle size of measured was found to be about 5 nm from TEM image (**Figure 3B**). The image is fully supported deconvoluted of SPR absorption spectra in to three Guassian peaks which shows the three different particles distribution pattern. SEM micrograph and EDX of PDDA treated cotton fiber and SNDC fiber (**Figure 4**) clearly reveals that small, spherical SNP are homogeneously deposited over the surface of cotton fibers. The EDX analysis (X-ray mapping) is also confirmed the amount and continuous distribution of SNPs (**Appendix A** and Supplementary Figure S1).

### Mechanism of SNDC Fabrication

The interaction of SNPs and cotton fibers can be understood through electrostatic interaction force. It is believed that cotton carry negative surface charges due to the presence of hydroxyl and carboxyl groups and PDDA, as a strong cationic polyelectrolyte, carries positive charges (Ribitsch et al., 1996; Grancaric et al., 2005; Tang et al., 2012). Consequently cotton fibers with negative charges have strong possibility to coat by PDDA with positive charges by electrostatic force. After reduction of Ag^+^ using citrate carried negative charge over the citrate stabilize SNPs (Tang et al., 2015; Peng et al., 2016). Thus citrate stabilized SNPs having negative charges can be adsorbed on the PDDA modified cotton fabrics with positive charges through electrostatic interaction. Moreover, it is found that PDDA unmodified SNDC fibers were less dark than modified SNDC fibers at same energy and time of microwave radiation during SNDC fabrication. Which confirm PDDA is playing a significant role in better interaction cotton fiber and SNPs. The mechanism of microwave assisted SNDC fabrication on cotton fibers is discussed as shown in (**Figure 1**).

### Antibacterial Susceptibility of Extracted SNPs Determination of Minimum Inhibitory Concentrations Determination of Minimum Bactericidal Concentration

**FIGURE 5:**
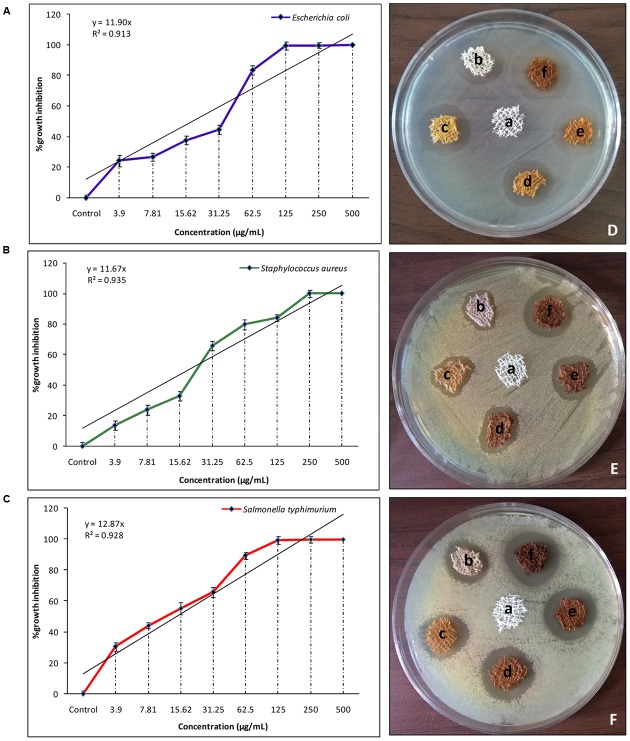
**Antibacterial activity of extracted SNPs and SNDC fibers (A)** Graphical representation of %growth inhibition over concentration against *E. Coli* **(B)** *S. aureus* **(C)** *S. typhimurium* (Data represent mean ± standard error of three replicates), **(D)** Zone of inhibition of fabricated SNDC fiber at different power of microwave radiation for 1 min exposure against *E. coli* **(E)** *S. aureus* **(F)** *S. typhimuriume* (a) Untreated cotton served as control (b) Treated with 100 W (c) 180 W (d) 300 W (e) 450 W (f) 600 W.

The *in vitro* antibacterial susceptibility was measured in terms of growth rates of *E. coli, Salmonella typhimurium* and *S. aureus* using turbidimetric growth analysis over a concentration range of NP (0–500 μg/mL). The MICs of the NPs against all the three strains are represented in (**Table 1**; **Figures 5A–C**) with percentage growth inhibition curve against concentration range along with a linear regression coefficient between the two plotted parameters. All the three strains exhibited a significant correlation between concentration range and % growth inhibition with a maximum *R*^2^ = 0.935 for *S. aureus*, followed by *Salmonella typhimurium* and *E. coli* with *R*^2^ values equal to 0.928 and 0.913 respectively (**Figures 5A–C**). SNPs showed better efficacy against Gram negative bacteria than Gram positive bacteria (Pandey et al., 2014). The results are in agreement with the previously reported findings (Kim et al., 2007; Jung et al., 2008; Ruparelia et al., 2008). This could be attributed to the cell membrane structures possessing the different amount of lipid and peptidoglycan layer (Mishra et al., 2016b). Gram positive bacteria with high peptidoglycan may prevent the action of NPs across bacterial cell wall (Feng et al., 2000; Pal et al., 2007).

**Table 1:** Determination of minimum inhibitory concentration (MICs) of extracted SNPs from SNDC fibers against selected bacterial pathogens (μg/mL).

Selected parameters	Antibacterial efficacy of SNPs against selected bacterial pathogens (μg/mL)
	*E. coli* (MTCC-723)	*S. aureus* (MTCC-737)	*S. typhimurium* (MTCC-98)
MICs	42.5 ± 1.33	125 ± 2.12	38.5 ± 0.93
IC_50_	31.2 ± 0.58	72.4 ± 1.91	25.3 ± 0.37
MBC	62.5 ± 1.89	158 ± 3.19	62.5 ± 1.89

### Flowcytometric Analysis for Plasma Membrane Permeabilization

**FIGURE 6:**
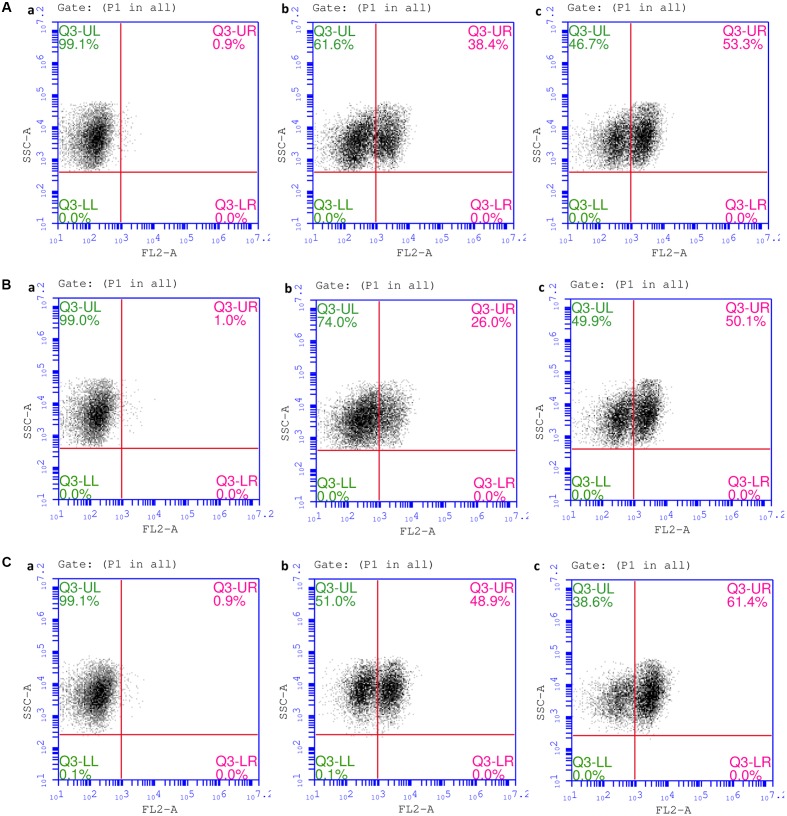
**Lesion on plasma membrane of selected pathogenic bacteria using different treatments of extracted SNPs from SNDC fibers showing sequence density plots with PI-stained cells (A)** *E. Coli* **(B)** *S. aureus* **(C)** *S. typhimurium*, (a) Untreated control cells, (b) Treated with 15.62 μg/mL of SNPs (c) Treated with 31.25 μg/mL of SNPs.

The effects of NPs over the plasma membrane integrity of bacterial strains were evaluated in terms of PI internalization through flowcytometry and protein leakage analysis. Two median doses 15 and 30 μg/L was selected from the specified concentration range and evaluated the role of SNPs in affecting membrane permeability. A dose dependent depletion in cell survival % in all three strains as reflected through PI influx. *E. coli* over treatment with a higher concentration of NPs revealed ∼60.1% of cell death and lower concentration resulted in ∼28% cell death (**Figure 6**). Furthermore, a higher dose of NPs resulted in PI internalization in ∼61 and 53% of *Salmonella* and *S. aureus* respectively; and, a lower dose of NPs resulted in ∼49 and 38% of cell death of two bacterial strains respectively (**Figures 6A–C**).

**FIGURE 7:**
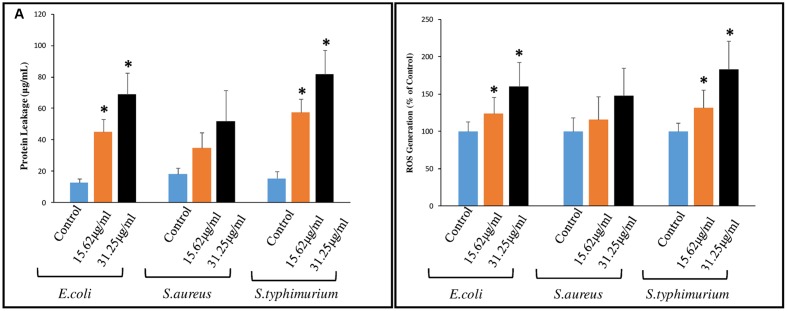
**Evaluation of membrane disruption by (A)** Protein leakage and **(B)** Relative fluorescence intensity showing cellular ROS formation potential of SNPs. Data are represented as Mean ± SEM, ^∗^*p* < 0.05 compare to control.

Comparative overlay histogram sequence density plots clearly revealed that SNPs could induce dose dependent membrane permeability in bacterial cells (**Appendix A** and Supplementary Figure S2). The membrane permeability could further facilitate SNPs penetration in bacterial cells and result in protein dysfunction (Hsueh et al., 2015). Protein leakage analysis further confirmed the loss of membrane integrity over NP-exposure (**Figure 7A**). A dose dependent increase in protein concentration was observed in culture supernatant of all the three bacterial strains as compared to control groups. The observations made through spectrophotometer and protein leakage analysis were found to be in line with the anti-bacterial susceptibility assay and previous research findings (Chakraborti et al., 2014). The bactericidal effect of SNPs was further studied in terms of Reactive Oxygen Species generation. **Figure 7B** reveal magnitude of ROS formation in terms of fluorescent counts due to DCA formation in bacterial cells exposed to SNPs. A dose dependent increase in ROS was observed against all bacterial species. *E. coli* and *S. typhimurium* showed a significant increase in oxidative stress level both at 15.62 μg/mL and 31.25 μg/mL, however, data remained insignificant in case of *S. aureus.* Although exact mechanism involving antibacterial efficacy of SNPs have not been clearly revealed ROS and free radicals have been variously documented to incur antibacterial potential to NPs (Sen et al., 2013; Dakal et al., 2016). The binding of Ag^+^ over the microbial cell membrane induces cytotoxicity which further inhibits mitochondrial respiratory chain enzymes (Blecher and Friedman, 2012) and disrupts electron transport chain by uncoupling oxidative phosphorylation (Belluco et al., 2016) subsequently leading to cell death.

### Antibacterial Efficacy of SNPs Decorated Cotton Using disk Diffusion Assay

Zone of inhibition of SNDC fibers against some gram-positive (*S. aureus*) and gram-negative bacteria (*E. coli* and *Salmonella typhimurium*) strains are shown in (**Figure 5**).

The synthesized samples size (0.5 cm x 0.5 cm) are labeled as (a) Control (PDDA untreated cotton) and (b) treated with 100 W (c) 180 W (d) 300 W (e) 450 W (f) 600 W SNDC fiber at for 1 min exposure. Radiant zones of inhibition were observed due to SNPs loading dependency against tested bacterial pathogens. The results are clearly shown in (**Figures 5D–F**).

### Filtration Efficacy of Silver NPs Decorated Cotton on *E. coli*

**FIGURE 8:**
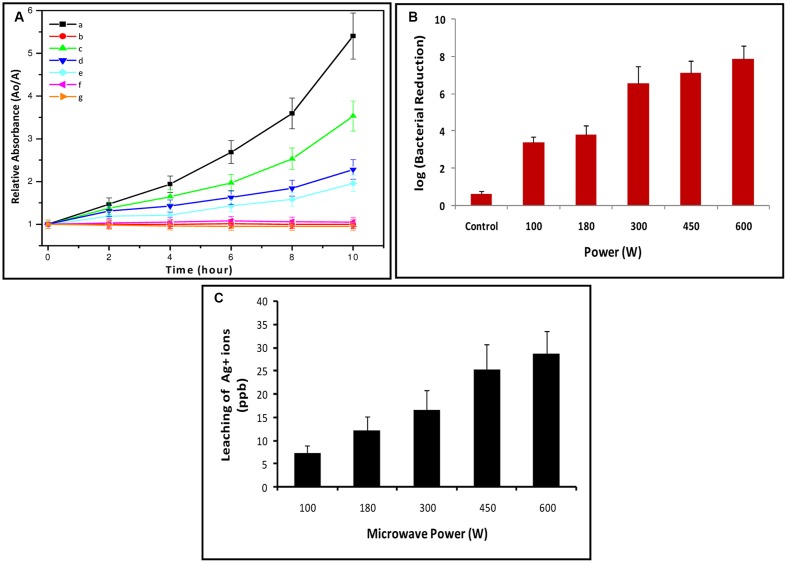
**(A)** Relative absorbance after each 2 h interval of *E. coli* permeated with different SNDC fibers at 492 nm. (a) Positive control (without SNPs in cotton) (b) Negative control (without bacteria) (c) Treated with 100 (d) 180 (e) 300 (f) 450 and (g) 600 W, **(B)** Log reduction of *E. coli* (1 × 10^8^ CFU/mL) count after permeation through SNDC sheets. **(C)** Leaching of Ag^+^ obtained in the effluent measured by ICPMS.

The SNDC fibers provides suitable and effective bactericidal activity as model *E. coli* suspensions were poured through the 0.4 cm thick dry consolidated SNDC fibers sheet which was put over filter paper. The average percolation time for 100 mL of bacterial suspension was 8 min. Some *E. coli* were retained in the cotton filter, but most of them passed through and were isolated from the effluent by centrifugation and analyzed for viability. The qualitative re-growth experiments in MHB showed inactivation of bacteria at the highest silver concentration (**Figure 8A**) shows the exponential growth curve in the positive control sample (undecorated cotton or without SNPs in cotton). While the negative control sample (without bacteria) shows no growth. The bacteria growth after percolation through the SNDC fibers was almost completely deactivated for the cotton with the highest SNPs content (microwave irradiation 100–600 W for 1 min). The lower SNPs containing cotton showed a maximum bacterial growth reduction in comparing with the positive control. To check further the bactericidal effectiveness of SNDC fibers, the isolated effluent bacteria was added to NA plates after passing through the cotton. The plate count experiments showed maximum log 7.8 reductions of viable *E. coli* in the effluent, as compared to the initial concentration of bacteria (10^8^ CFU/mL) (**Figure 8B**). The positive control also showed a reduction in bacteria by log 0.59, most likely due to some bacteria remaining on the surfaces of cotton fiber. The SNDC fibers prepared within one min with different microwave power at 100, 180, 300, 450, and 600 W, responsible to log bacterial reduction log 3.3 (±0.09), log 3.8 (±0.11), log 6.5 (±0.19), log 7.1 (±0.2) and log 7.8 (±0.22) respectively. This clear difference was observed in reduction potency of bacteria due to increasing concentration of SNPs over cotton that are also supported by UV-VIS reflectance spectra.

The antimicrobial nature of a few SNPs is believed to be only through the contact killing mechanism, which contributes an even greater potential lethality when bacteria come in contact with them (Li et al., 2009; Srinivasan et al., 2013). However, in this work contact mode are preferred for the testing of the bactericidal effectiveness of the SNDC fibers, we have approved model bacterial suspension through an SNDC fiber sheet and analyze bacterial viability in effluent water. In this purification, SNDC fibers was not used for removal of *E. coli* from effluent by filtration but rather the deactivation of bacteria as they percolate through the highly porous SNDC fibers structure. The large pore size of the cotton and base as filter paper allows good retention and contact killing of bacteria as well as allow reasonable balance flow by gravity, without the need for pressure or suction. Due to possible human health effects from silver exposure, we analyzed the silver content of the effluent water. We used centrifugation to separate bacteria from the silver leached out from the SNDC sheet. Highest amount of silver content of ∼28.75 ppb was observed (SNDC prepared at 600 W for 1 min) using ICP-MS (**Figure 8C**). The amount of silver leaching from the SNDC filter thus meets the US-EPA guideline for drinking water of less than 100 ppb (Dankovich, 2014). This product can be easily prepared at home for multipurpose point of use decontamination of water, surface sterilization of medical equipment and human hand.

## Conclusion

Microwave assisted SNDC fiber samples have been prepared through *in situ*, rapid, convenient, environmental friendly and cost effective method. The SNPs were well dispersed and stabilized on the surface of the cotton fiber. The fabricated NPs were found to be effective against both Gram positive and Gram negative bacteria as revealed through broth microdilution and disk diffusion assay. Cell death could also be credited to ROS generation. Furthermore, the PI influx and protein leakage studied also indicated toward membrane damage incurred due to NPs exposure. Thus the results of contact killing of microbes indicate that it can be applied as a point of use surface disinfectant of wounds, preservative as well as for the development of an effective filter for drinking water in developing countries.

## Author Contributions

AB, RG, and RM conceived and designed the experiments; KU, SCS, AB, AS, and MS carried out the synthesis, characterization and analysis of silver nanoparticles decorated cotton (SNDC) fibers; AB, RM, and SS performed the antibacterial experiments; VM, RM, and AB and performed and analyzed ROS and flowcytometry data. AB, RG, RM, VM, and SCS wrote the paper. All authors have read and approved the final manuscript.

## Conflict of Interest Statement

The authors declare that the research was conducted in the absence of any commercial or financial relationships that could be construed as a potential conflict of interest.

## Appendix A. Supplementary data

Supplementary data SEM X-ray mapping of normal and SNDC fibers as well as their corresponding EDX maps. Comparative overlay histogram sequence density plots showing lesion on plasma membrane of selected bacterial pathogens using different treatments of extracted SNPs from SNDC fibers associated with this article can be found in the online version.
