# Supplementary material for: Power and Time Dependent Microwave Assisted Fabrication of Silver Nanoparticles Decorated Cotton (SNDC) Fibers for Bacterial Decontamination
Source: Front Microbiol. 2017 Mar 3;8:330. doi: 10.3389/fmicb.2017.00330 (PMC5334347; doi:10.3389/fmicb.2017.00330)
Supplement: Supplementary file 1 [file Data_Sheet_1.DOC]

**Power and time dependent microwave assisted fabrication of silver nanoparticles decorated cotton (SNDC) fibers for bacterial decontamination**

*Abhishek K. Bhardwaj^1,2*^, Abhishek Shukla^2^, Rohit K. Mishra^3*^, S. C. Singh^2,4^, Vani Mishra^3^, K. N. Uttam^2^, Mohan P. Singh^5^, Shivesh Sharma^3^* and *R. Gopal^3*^*

^1^Centre for Environmental Science, University of Allahabad, Allahabad-211002, India

^2^Laser Spectroscopy and Nanomaterials Lab., Dept. of Physics, University of Allahabad, Allahabad-211002, India

^3^Centre for Medical Diagnostic and Research, Motilal Nehru National Institute of Technology, Allahabad-211004, India

**^4^**High Intensity Femtosecond Laser Laboratory, The Institute of Optics, University of Rochester, Rochester, NY 14627,USA.

^5^Centre of Biotechnology, University of Allahabad, Allahabad-211002, India

**^*^Corresponding Authors Email:** bhardwajak87@gmail.com; rohit_ernet@yahoo.co.in; [rgopal.prof@gmail.com](mailto:rgopal.prof@gmail.com)

**Suppl. FIGURE 1** Scanning electron micrograph (SEM) X-ray mapping of **[A]** Normal cotton fibers **[B]** SNDC fibers as well as their corresponding EDX maps

**Suppl. FIGURE 2** Comparative overlay histogram sequence density plots showing lesion on plasma membrane of selected bacterial pathogens using different treatments of extracted SNPs from SNDC fibers [A] *E. Coli* [B] *S. aureus* [C] *S. typhimurium,* (a) Untreated control cells (b) Treated with 15.62 µg/mL (c)Treated with 31.25 µg/mL
